# Improved Bioavailability and Antitumor Effect of Docetaxel by TPGS Modified Proniosomes: *In Vitro* and *In Vivo* Evaluations

**DOI:** 10.1038/srep43372

**Published:** 2017-03-07

**Authors:** Helong Liu, Liangxing Tu, Yongxin Zhou, Zefang Dang, Luting Wang, Junfeng Du, Jianfang Feng, Kaili Hu

**Affiliations:** 1 Murad Research Center for Modernized Chinese Medicine, Shanghai University of Traditional Chinese Medicine, Shanghai, 201203, People’s Republic of China

## Abstract

A novel oral drug delivery system, TPGS modified docetaxel proniosomes (DTX-TPGS-PNs), was designed to enhance the oral bioavailability and antitumor efficiency of the poorly water-soluble drug docetaxel. DTX-TPGS-PN niosomes were 93 ± 6.5 nm in size, −18.53 ± 1.65 mV in zeta potential and exhibited spherical morphology, with an encapsulation efficiency of 97.31 ± 0.60%. The system showed sustained release in both simulated gastric and intestinal fluid. The results of caco-2 monolayer, everted gut sac model and improved single-pass intestinal perfusion model transport studies showed that DTX-TPGS-PN niosomes could significantly improve the absorption of DTX. The pharmacokinetics study suggested the absolute bioavailability of DTX-TPGS-PN niosomes were 7.3 times that of DTX solution. In addition, a higher antitumor efficacy than DTX solution was demonstrated in MCF-7 and MDA-MB-231 cells *in vitro* and in MCF-7 tumor-bearing mice model *in vivo*. Our results demonstrated DTX-TPGS-PN is promising in enhancing the bioavailability and efficiency of poorly water-soluble drug DTX, and the potential of proniosomes as stable precursors for oral drug delivery.

Drug delivery system has been investigated extensively for their important roles in improving bioavailability of the poorly water-soluble drug. Niosomes, as one of which, has received great attention for its similar structure but higher stability and lower cost compared to liposomes. Its closed bilayer structure was formed from the self-assembly of non-ionic surfactant in aqueous media^1^,^2^,^3^. *In vivo* studies showed that niosomes behave like liposomes, prolonging the circulation time of the entrapped drug and altering its organ distribution as well as metabolic stability^4^. The problems of the physical stability of niosomes were not alleviated, until proniosomes of solid state was introduced, which is produced with non-ionic surfactants and could be hydrated immediately before use to form a niosomes suspension suitable for administration. Proniosomes may minimize the previously mentioned physical stability problems. Besides, the convenience for the storage, transportation would make proniosomes a promising industrial product.

D-alpha-tocopheryl polyethylene glycol succinate (TPGS), a water-soluble polyethylene glycol (PEG) derivative of natural-source vitamin E, has an amphiphilic structure comprising of a hydrophilic polar head group (PEG) and a lipophilic alkyl tail (tocopherol succinate). Due to the bulky structure and large surface area, TPGS is an excellent emulsifier, solubilizer and bioavailability enhancer for hydrophobic drugs^5^,^6^, and can increase the solubility of drugs including taxanes, steroids and antibiotics. In addition, TPGS can inhibit P-glycoprotein (P-gp) excretion to improve drug permeability through cell membranes, thus reducing P-gp-mediated multidrug resistance (MDR) in cancer cells, by which it can further enhance absorption and increase the cytotoxicity and oral bioavailability of anticancer drugs^7^,^8^. Besides, TPGS is an FDA approved pharmaceutically safe adjuvant which is widely used for the delivery of anticancer drugs^6^.

Docetaxel (DTX) is a widely used anticancer taxane for the treatment of malignant breast, ovarian, and lung tumors^9^. With a similar structure to paclitaxel, DTX also bind to tubulin. However, the affinity of DTX is 1.9-fold higher than that of paclitaxel^10^. Because of its poor aqueous solubility, DTX is currently formulated as the marketed product Taxotere^®^, which contains ethanol and the nonionic surfactant Tween-80. However, Tween-80 has been shown to induce hypotension, tachycardia, a rise in histamine levels, and promote the generation of biologically active complement products^11^,^12^. Clinically, the Tween-80 formulation is known to cause severe allergic reactions and peripheral neuropathy^13^. Therefore, various formulations were developed to improve the solubility of DTX while avoiding the side effects.

Oral delivery was an attractive route to deliver therapeutics via nanoparticles, for it affording easy handling, high patient compliance, less stringent production conditions and lower costs^14^. As for oral delivery of DTX, the major problem is its low bioavailability, which in part is caused by the excretion effect of P-glycoprotein. Here, we developed a new oral drug delivery system of DTX, the TPGS modified proniosomes (DTX-TPGS-PN), to overcome the low permeability and poor oral bioavailability of DTX and avoid the side effects of Taxotere^®^. Characterization of DTX-TPGS-PN niosomes was performed by dynamic light scattering (DLS) particle sizer and transmission electron microscope (TEM). The *in vitro* Caco-2 cell model, *ex vivo* everted gut sac model and *in situ* improved single-pass intestinal perfusion model were used to investigate the effect of DTX-TPGS-PN niosomes on absorption of DTX. Then, the antitumor efficacy of DTX-TPGS-PN niosomes was studied *in vitro* by MCF-7 and MDA-MB-231 cells and *in vivo* by MCF-7 tumor-bearing nude mice.

## Results and Discussion

### Preparation and characterization of DTX-TPGS-PN

**Figure 1:**
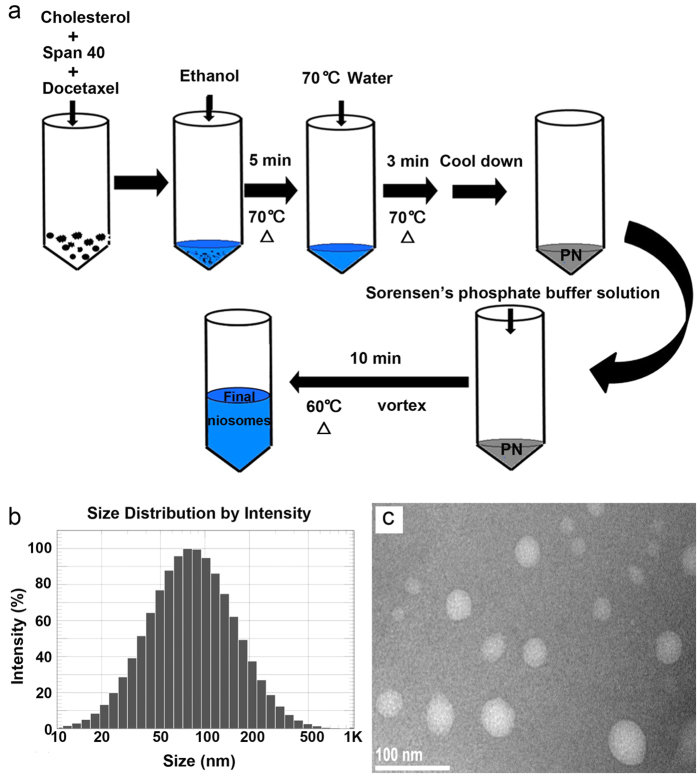
Preparation and characterization of DTX-TPGS-PN niosomes. (**a**) Schematic diagram for the preparation of proniosomes and niosomes. (**b**) The size distribution of DTX-TPGS-PN niosomes measured by DLS. (**c**) The morphology of DTX-TPGS-PN niosomes observed by TEM.

Proniosomes were prepared by the method reported by Mahmoud Mokhtar *et al*. with some modifications (Fig. 1a)^15^. The prepared DTX-TPGS-PN niosomes had average diameters of around 90 nm (Fig. 1b) and a zeta potential of −18.53 ± 1.65 mV. TEM data collected for DTX-TPGS-PN niosomes demonstrated that the nano-sized particles are spherical in shape and are uniformly distributed, however, with a smaller diameter of around 40 nm (Fig. 1c). It could be explained by that the nanoparticles measured by DLS were hydrated whereas those by TEM was dehydrated^16^. The DTX-TPGS-PN niosomes displayed a strong negative zeta potential, which is good for the stability of the niosomes against aggregation and fusion^17^. The encapsulation efficiency were 97.31 ± 0.60% for DTX-TPGS-PN niosomes and 91.50 ± 2.17% for DTX-PN niosomes. Indeed, it has been reported that TPGS is an efficient emulsifier that could realize 100% encapsulation efficiency (EE) of paclitaxel in poly(lactic-co-glycolic acid) (PLGA) NPs^18^. As we all known, particle size plays an important role on clearance and tumor uptake of nanoparticles^19^. Smaller particles (50–300 nm) are commonly removed from the circulation relatively slowly^20^. So the size distribution is generally controlled around 100 nm. The *in vitro* release results showed that both DTX-TPGS-PN and DTX-PN niosomes totally released in 12 h and 8 h in simulated intestinal fluid (SIF) and simulated gastrointestinal fluids (SGF), respectively.

### Transport of DTX-TPGS-PN niosomes across the Caco-2 cell monolayer

**Figure 2:**
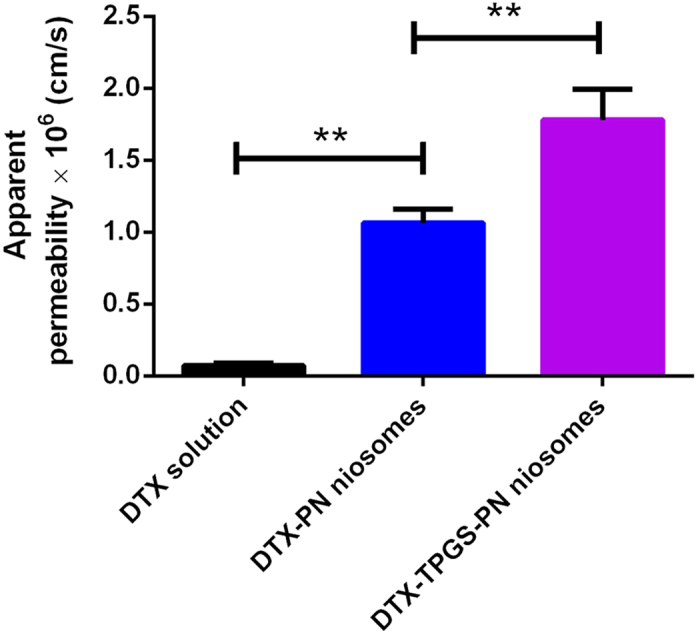
The apparent permeability of DTX on Caco-2 cell monolayer by DTX solution, DTX-PN and DTX-TPGS-PN niosomes. **P<0.05, *n* = 3.

The effect of DTX-TPGS-PN and DTX-PN niosomes on transport of DTX across the Caco-2 monolayer was investigated, and comparing to DTX solution. The Caco-2 cell monolayer has a transepithelial electrical resistance of 488 ± 73 Ω·cm^2^. Figure 2 showed that compared to DTX solution, the DTX-PN niosomes had higher apparent permeability coefficient (P_app_), and once modified with TPGS, the P_app_ of DTX-TPGS-PN niosomes was significantly higher than the DTX-PN niosomes. Although is often used to study intestinal transport, one disadvantage of the Caco-2 monolayer is its impermeability to hydrophilic or paracellular transport chemicals. Therefore it’s a better model for colonic tissue permeability study with restricting reliability for the small intestinal tissue permeability study^14^. And because there are only epithelial cells in the intestinal epithelium, in contrast to many other cell types including mucosal cells and microfold cells (M-cells) in the real intestinal, we further used everted gut sac model and *in situ* improved single-pass intestinal perfusion model to further verify the results.

### Everted gut sac model study

**Figure 3:**
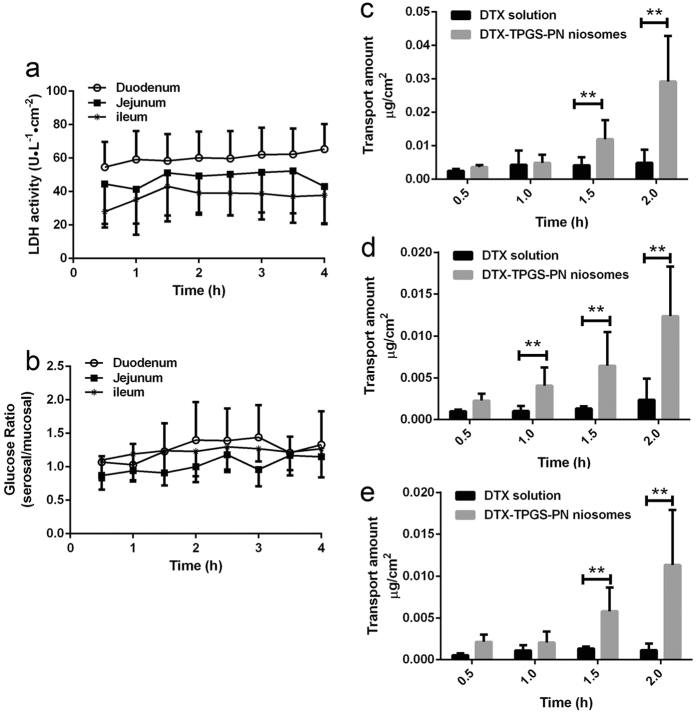
The absorption of DTX in everted gut sac model. LDH release (**a**) and glucose transport (**b**) of duodenum, jejunum and ileum in the everted intestinal sac model (*n* = 4 and *n = *5, respectively). The absorption of DTX by DTX-TPGS-PN niosomes and DTX solution in the duodenum (**c**), jejunum (**d**) and ileum (**e**) in the everted intestinal sac model. **P<0.05, *n* = 4.

Lactate dehydrogenase (LDH) is an intracellular enzyme, detected following damage to cell membranes, to be used as a biochemical marker of intestinal wall damage. The LDH release results in everted gut sac model are shown in Fig. 3a. At 0 h, LDH activity in the incubation medium of duodenum was 54.59 U/L/cm^2^. This was probably due to the surgical procedures when excising and everting the gut sac. Compared to the start, no significant differences of LDH activity at 1 h, 2 h, 3 h or 3.5 h were found in duodenum, and neither did in jejunum and ileum. It was concluded that the everted gut sacs maintained their viability during the period of the experiment and consequently this model was suitable for testing the DTX transport.

The concentration ratios of glucose between serosal side and mucosal side are shown in Fig. 3b. It was obvious that there were no significant differences on the ratios during the whole experiment process, indicating the tissue of the gut sac was viable and well-functioning.

The time course of transport of DTX across small intestinal segments is illustrated in Fig. 3c–e. There were no significant regional differences in intestinal absorption of DTX solution. The total drug uptake is: ileum < jejunum < duodenum (0.0015, 0.0024, and 0.0049 ug/cm^2^, respectively), which may probably due to physiological differences in expression of P-gp among them. DTX is a substrate of P-gp and the expression of P-gp in the ileum was 2.31-fold higher than that in the jejunum of rat. These may cause more DTX retained in duodenum than in ileum or jejunum. For each segment, drug amount was accumulated along with the time increasing. And significant improvement of DTX absorption by DTX-TPGS-PN niosomes was observed (p < 0.05) from 1.5 h to 2 h, indicating DTX-TPGS-PN niosomes may have better permeability than DTX. It might be attributed to the combination of the small particle size of DTX-TPGS-PN niosomes which allows efficient uptake in the intestine and the TPGS which could enhance the bioavailability by inhibiting P-gp activity^5^,^21^.

### Improved single-pass intestinal perfusion model study

Improved single-pass intestinal perfusion model provides a functional intestinal barrier and conditions that closely mimic the normal physiological state following oral administration in terms of providing an intact blood supply^22^. And a high correlation between rat and human P_eff_ values has also been found^23^. So the improved single-pass intestinal perfusion model was also used to verify the intestinal absorption of the DTX-TPGS-PN niosomes.

**Figure 4:**
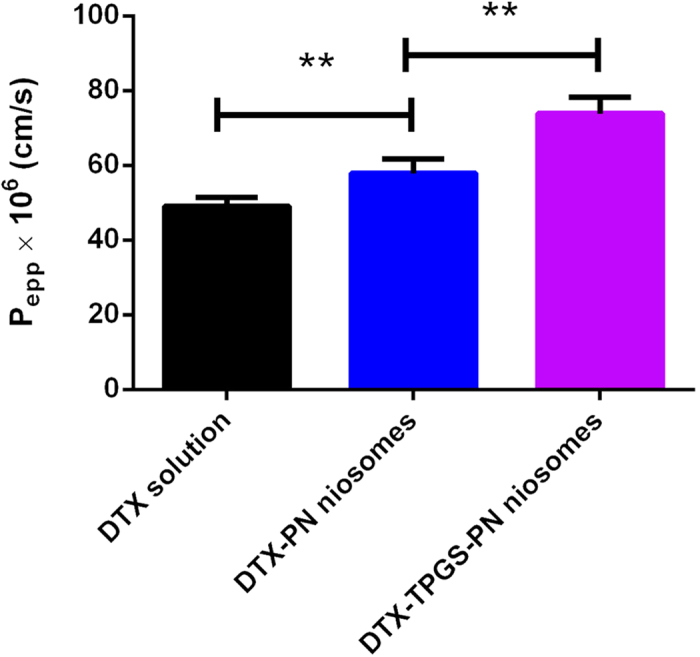
The P_eff_ of DTX solution, DTX-PN and DTX-TPGS-PN niosomes in the single-pass intestinal perfusion model (*n* = 4, **P<0.05).

Figure 4 showed the DTX permeability of DTX solution, DTX-PN and DTX-TPGS-PN niosomes in the improved *in situ* perfusion model. Compared to the 48.7 × 10^−6^ cm/s for DTX solution, the P_eff_ value for DTX-PN and DTX-TPGS-PN niosomes were improved to 57.89 × 10^−6^ cm/s and 73.84 × 10^−6^ cm/s, which indicated the niosomes improved the bioavailability of DTX. The P_eff_ value of DTX-TPGS-PN group was significantly higher than the DTX group, demonstrated the DTX-TPGS-PN niosomes do have an enhancing effect on the permeability of DTX. The P_eff_ value of DTX-TPGS-PN group was also significantly higher than the DTX-PN group.

While, comparing with single-pass perfusion model, the improved single-pass perfusion model conducted with the whole small intestine instead of a part of intestine was proved feasible and had the advantage of maintaining better physiological conditions of the intestine^24^. Each model has its limitations but an association of them would provide us a better understanding of DTX absorption. By comparing the results of Caco-2 monolayer model, everted gut sac model and single-pass intestinal perfusion model, we can draw a conclusion that DTX-TPGS-PN niosomes have an improved permeability in intestine than DTX solution. And this may be explained by the advantages of niosomes with small size and the inhibition effect of P-glycoprotein by TPGS. It is well known that DTX is a P-gp substrate and the oral absorption is partly affected by P-gp activity. TPGS with P-gp inhibition ability may increase the oral absorption as well as tumor cell toxicity.

### Pharmacokinetic studies of DTX-TPGS-NP

**Figure 5:**
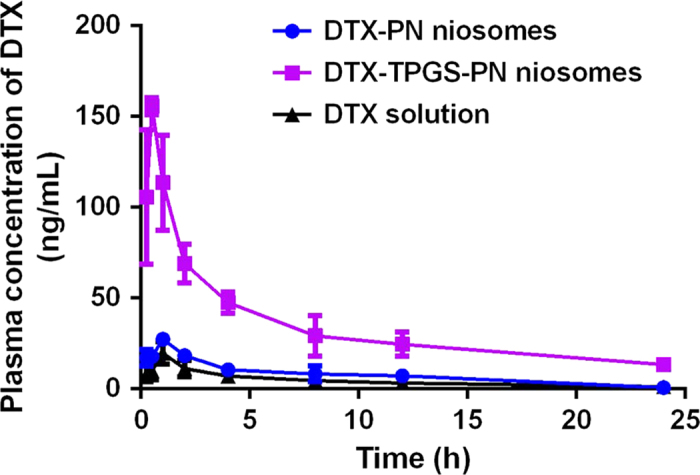
The concentration-time curves of DTX in plasma followed by oral administration of DTX-TPGS-PN, DTX-PN niosomes and DTX solution in rats at a dose of 20 mg·kg^−1^ DTX (*n* = 3).

The blood concentration–time curves of DTX after p.o. administration of DTX solution, DTX-PN and DTX-TPGS-PN niosomes in rats were showed in Fig. 5.

As showed in Table 1, the area under the curve (AUC) was significantly increased from 109.04 ± 17.74 ng/mL·h for DTX solution to 184.70 ± 13.05 ng/mL·h for DTX-PN niosomes and 796.44 ± 83.93 ng/mL·h for DTX-TPGS-PN niosomes, which corresponds to a significantly increase of the oral bioavailability from 2.32% to 3.93% and 16.94%, respectively. On the other hand, C_max_ for DTX solution was 19.83 ± 6.30 ng/mL, which is statistically lower than 27.43 ± 1.53 ng/mL for DTX-PN and 155.67 ± 4.73 ng/mL for DTX-TPGS-PN. While the T_max_ for DTX solution and DTX-PN niosomes was reduced from 1 h to 0.5 h for DTX-TPGS-PN niosomes. The enhanced and faster absorption of DTX-TPGS-PN niosomes in intestine was correlated with the *in vitro* absorption results. A particle size of around 100 nm and incorporation of TPGS, which works as P-gp excretion inhibitor, results in efficient uptake and a higher C_max_ of DTX-TPGS-PN niosomes. Along with the everted gut sac model and the single-pass intestinal perfusion model, improved bioavailability of DTX by DTX-TPGS-PN niosomes was proved.

### *In vitro* cytotoxicity of DTX-TPGS-NP

**Figure 6:**
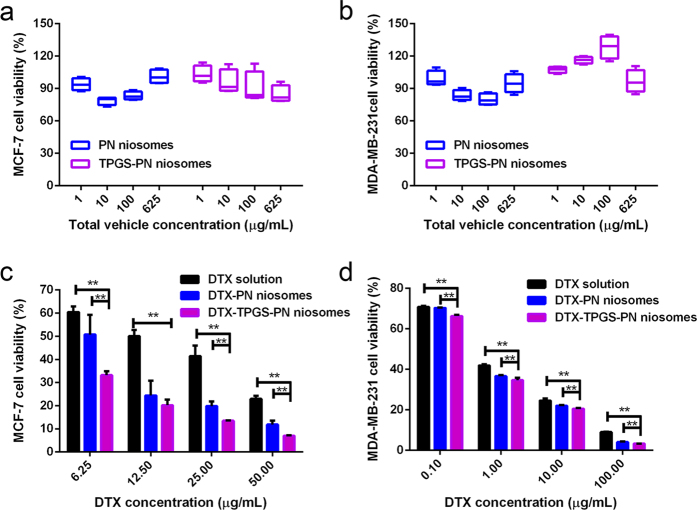
*In vitro* cytotoxicity of PN, TPGS-PN, DTX-TPGS-PN, DTX-PN niosomes and DTX solution in MCF-7 (a,c) and MDA-MB-231 (b,d) cells (Mean ± SD, *n* = 3–4, **P < 0.05).

Several studies reported that DTX has inhibitory effects on breast cancer cell lines such as MCF-7 and MDA-MB-231^25^,^26^. The cell viability was evaluated by the CCK-8 assay. DTX solution, DTX-PN and DTX-TPGS-PN showed a dose-dependent cytotoxicity toward both MCF-7 and MDA-MB-231 cells (Fig. 6c and d). The IC_50_ of DTX solution, DTX-PN and DTX-TPGS-PN niosomes were 0.715 μg/mL, 0.508 μg/mL, 0.378 μg/mL in MCF-7 cells and 12.337 μg/mL, 5.469 μg/mL, 2.542 μg/mL in MDA-MB-231 cells, respectively. It has been reported that TPGS could lead to a higher cellular uptake and therapeutic effects in cancer cells^5^,^6^,^27^. Our study also proved the DTX-TPGS-PN niosomes enhanced the cytotoxicity of DTX compared with DTX solution and DTX-PN niosomes on MCF-7 and MDA-MB-231cell lines. The cytotoxic effect of blank PN and TPGS-PN niosomes were also evaluated in the two cell lines (Fig. 6a and b). In a wide range of vehicle concentrations (~625 μg/ml), the PN and TPGS-PN niosomes did not show significant cytotoxicity toward the cells, indicating that blank PN and TPGS-PN niosomes are nontoxic to tumor cells.

### Antitumor effect of DTX-TPGS-PN in MCF-7 cancer-bearing mice

**Figure 7:**
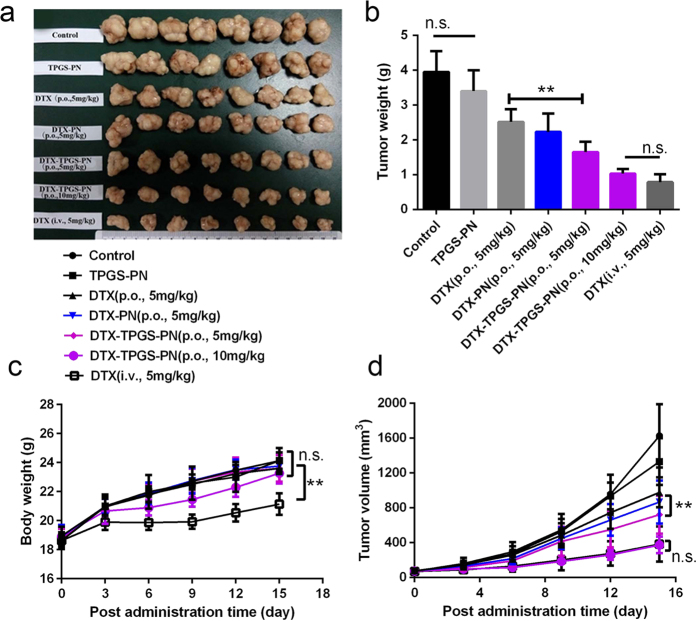
*In vivo* antitumor effect of various formulations on MCF-7-bearing nude mice. Photographs of the tumors (**a**), tumor weigh (**b**), body weight (**c**), and tumor volume (**d**) after different treatments (*n *= 8, **P < 0.05).

The antitumor effect of DTX-PN (p.o., DTX 5 mg/kg) and DTX-TPGS-PN niosomes (p.o., DTX 5 mg/kg, 10 mg/kg) on MCF-7 bearing nude mice were shown in Fig. 7 compared with NS (p.o., control group), TPGS-PN (p.o., the same dose of vehicle as DTX-TPGS-PN niosomes at 5 mg/kg DTX), DTX (p.o., 5 mg/kg), and marketed DTX injection (i.v., 5 mg/kg). NS and vehicle TPGS-NP treatments had no substantial effect on the tumor growth, and the tumor volumes increased rapidly. The treatment with DTX (p.o., 5 mg/kg) and DTX-PN (p.o., 5 mg/kg) was effective in tumor regression to some extent, but neither have advantage when was comparable to the i.v. DTX injection or DTX-TPGS-PN niosomes, which were more efficacious in tumor reduction (Fig. 7d). Figure 7b showed the masses of the tumors were with the same tendency. The data confirmed the anti-tumor efficacy of i.v. DTX injection and p.o. DTX-TPGS-PN niosomes. This may be contributed to the enhanced absorption of DTX-TPGS-PN niosomes. The role of TPGS is necessary which not only enhanced the absorption of DTX to inhibit the excretion of P-gp but also served as an apoptosis inducer^28^. It has been widely demonstrated that polymers with a MW >40 kDa and nanoparticles between 10 and 200 nm in size display reduced renal clearance (enhanced PK), and are able to migrate through open malignant neovasculature and accumulate in tumors via the enhanced permeability and retention effect^29^. The EPR effect of nanoparticles may also results in the enhanced antitumor effect of DTX-TPGS-PN niosomes.

In addition, body weight was monitored through the study to assay the possible toxicity effect caused by DTX-TPGS-PN niosomes (Fig. 7c). Upon statistical analysis, there was no significant change of body weight during the whole experimental period, excepting the DTX injection group. The body weight reduction of the DTX injection group maybe caused by the systemic toxicity cause by the high concentration of DTX in plasma. It has been reported that Taxotere may cause significant stress and neutropenia to the mice at 40 mg/kg DTX^30^. Body weight in mice of TPGS-PN was almost as the same as control group, which may explained as the safety of the TPGS-PN. Although the body weight increase was a little lower compared to the control group, no significant differences was found, indicating that the oral delivery of DTX-TPGS-PN was safe.

## Conclusion

A novel oral delivery system of docetaxel (DTX-TPGS-PN) was developed to improve the oral bioavailability of docetaxel. The prepared DTX-TPGS-PN niosomes had a diameter of 93 ± 6.5 nm and a high encapsulation efficiency of 97.31 ± 0.60%. The Caco-2 monolayer uptake study, the everted gut sac model experiment and improved single-pass intestinal perfusion model study showed that DTX-TPGS-PN niosomes can significantly improve the absorption of DTX compared with DTX solution. The pharmacokinetics study results suggest increased absorption *in vivo* with a 6.3 times increased absolute bioavailability of DTX-TPGS-PN comparing to DTX solution. The cytotoxicity of DTX-TPGS-PN in MCF-7 and MDA-MB-231cells was also significantly enhanced. In addition, antitumor effect in MCF-7 tumor-bearing mice suggested DTX-TPGS-PN has greater antitumor activity than DTX solution. Above results suggest DTX-TPGS-PN are valuable as a promising oral drug delivery system to enhance the oral delivery of DTX.

## Materials and Methods

### Materials and animals

Docetaxel was purchased from Shanghai Techwell Biopharmaceutical Co. Ltd (Shanghai, China). D-alpha-tocopheryl polyethylene glycol 1000 succinate (TPGS) was purchased from Sigma–Aldrich Co. (MO, USA). LDH commercial test kit was supplied by Jiancheng Biotech Institute (Nanjing, China). Glucose commercial test kit was purchased from Rongsheng Biotech Co. Ltd (Shanghai, China). Cell counting kit-8 was from Dojindo Laboratories (Kumamoto, Japan). Dulbecco’s modified Eagle medium, fetal bovine serum, nonessential amino acid, penicillin, and streptomycin were from Invitrogen Life Technologies (Carlsbad, CA). All other chemicals and solvents were of analytical reagent grade.

Male Sprague Dawley rats of 200–220 g weight and female nude mice of 4–6 weeks were from Shanghai Super B&K Laboratory Animal Corporation Ltd (Shanghai, China) and maintained at 22 °C ± 2 °C on a 12–hour light–dark cycle with access to food and water ad libitum. The animals used for the experiment were treated according to the protocols evaluated and approved by the ethical committee of Shanghai University of Traditional Chinese Medicine (Shanghai, China). Caco-2 cells were a kind gift from Professor Jianxin Wang (School of Pharmacy, Fudan University, Shanghai, China). MCF-7 cells were obtained from Type Culture Collection of the Chinese Academy of Sciences (Shanghai, China).

### Preparation of proniosomes (PN)

In brief, 0.300 g Span 40, 0.097 g cholesterol and 0.004 g DTX were dispersed in 800 mg ethanol and heated in a water bath at 70 °C for 5 min, then 0.32 mL deionized distilled water of the same temperature was added in and incubated at 70 °C for 3 min. The system was cooled down and white creamy proniosomal gel was obtained at room temperature. All formulations were hydrated to niosomes before use in this study.

### Preparation of niosomes

For the preparation of DTX-TPGS-PN niosomes, 14 mL Sorensen’s phosphate buffer solution (pH 7.4, contains 0.015% TPGS, w/v) was added in the PN and heated in a water bath at 60 °C for 10 min, with vortexing for 3 times during the heating. The final volume was adjusted to 20 mL by the same buffer without TPGS. DTX-PN niosomes were prepared by the same method as DTX-TPGS-PN except that 14 mL Sorensen’s buffer solution without TPGS was used instead.

### Particle size and zeta potential

Size and zeta potentials measurements were performed at room temperature by a Nicomp Particle Sizing system (380ZLS, Santa Barbara, California, USA). Particle sizes were measured directly and zeta potentials were analyzed after being diluted 100 times with distilled water.

### Morphology

The morphology of DTX-TPGS-PN niosomes was observed by transmission electron microscope (TEM, H-6009IV, Hitachi, Japan). The samples were diluted with distilled water and negatively stained with phosphotungstic acid before observation.

### *In vitro* release

To investigate the release kinetics of DTX-TPGS-PN niosomes in different body fluids, freshly prepared niosomes were exposed to simulated gastrointestinal fluids (0.1% Tween-80, pH 1.2; SGF: 1 L contains 2 g sodium chloride, 3.2 g pepsin, 7 mL hydrochloric acid) or simulated intestinal fluid (0.1% Tween-80, pH 6.8; SIF:1 L contains 6.8 g monobasic potassium phosphate, 10 g pancreatin, 77 mL 0.2 N sodium hydroxide)^31^. The test tube each with 1 mL DTX-TPGS-PN niosomes solution was immersed in a water bath at 37 °C and gently shaken at 100 rpm. Six tubes were withdrawn at each predetermined time intervals, three of them were centrifuged at 12000 rpm for 20 min immediately and the supernatant was diluted 1:3 (v/v) with acetonitrile and followed by assay of HPLC, which is defined as the amount of released DTX in the media. The other 3 samples were directly diluted 1:3 (v/v) with acetonitrile and followed by assay of HPLC, which is defined as the total amount of DTX in the solution.

### Transport across the Caco-2 cell monolayer

Caco-2 cells were used as an *in vitro* model to evaluate the intestinal absorption of DTX-TPGS-PN niosomes. The cells were maintained in Dulbecco’s Modified Eagle’s Medium (DMEM, Gibco) with 10% (v/v) fetal bovine serum (FBS, Gibco), 2 mM glutamine, 100 units/mL penicillin and 100 μg/mL streptomycin, incubating at 37 °C with 5% CO_2_ and fully humidified conditions. Prior to the experiments, Caco-2 cells were seeded into MILLICELL-^®^PCF culture plate at 8 × 10^4^ cells/well. The culture media was added to the apical (0.4 mL) and basolateral (0.6 mL) side, and was replaced every two days for the first week and everyday thereafter^32^. Cells were incubated for 21–25 days until the transepithelial electrical resistance (EVOM voltohmmeter; Millipore) increased to around 500 Ω/cm^2^. Before the experiments, the monolayer in each well was washed three times with preheated 37 °C Hanks’ balanced salt solution. Then, 0.4 mL DTX solution, DTX-PN or DTX-TPGS-PN niosomes were added to the apical side with final DTX concentration of 40 μg/mL, and 0.6 mL Hanks’ balanced salt solution was added to the basolateral side. At 15 and 30 min after the incubation, 0.3 mL medium was taken from the basolateral side and replaced with preheated fresh Hanks’ balanced salt solution. Then, 0.1 mL of the samples were lyophilized to dry powder and re-dissolved by 0.1 mL acetonitrile for HPLC assay.

### Transport of DTX in the everted gut sac model

The everted gut sac was used as an *in vitro* model to study the intestinal absorption of the DTX-TPGS-PN niosomes. To prepare the gut sac, male SD rats fasted overnight (free access to water) were anesthetized and the intestinal segments of interest were identified (duodenum, starting 2 cm below the pylorus; jejunum, starting from 20 cm below the pylorus; ileum, starting 20 cm above ileocecal junction) and isolated^33^. Each intestinal segment was flushed with cold saline and immediately placed in 37 °C oxygenated (O_2_/CO_2_, 95%:5%) Kreb-Ringer’s buffer (glucose 7.78 mM, NaCl 133 mM, KCl 4.56 mM, NaH_2_PO_4_ 1.50 mM, MgCl_2_ 0.20 mM, NaHCO_3_ 16 mM, CaCl_2_ 3.33 mM, pH was adjusted to 7.0–7.2) solution. The underlying mesenterium and fat were carefully removed and the sacs were gently everted with a glass rod. One end of the segment was clamped and tied with a silk thread forming a sac, while the other end was attached to a sampler. After the blank solution (1 mL) was introducing into the everted sac (serosal side), a 10-cm-long everted gut sac was prepared. Each sac was individually placed in a 15 cm glass tube (containing oxygenated Kreb-Ringer’s buffer solution), which was incubated in a 37 °C water bath with 50 rpm shaking.

Four everted gut sacs of the duodenum, jejunum and ileum segment, respectively, were made to investigate the permeability of different intestine segments. The prepared sacs were pre-incubated for 5 min in oxygenated blank Kreb-Ringer’s buffer^24^. Then, sacs were incubated in 37 °C oxygenated Kreb-Ringer’s buffer solution, and DTX solution and DTX-TPGS-PN niosomes were added respectively. At each predetermined time, 0.1 mL of serosal fluid was aspirated from the gut sac and preheated blank buffer of same volume was added. After the experiment, the area of each sac was measured. Samples were kept at −20 °C before analysis by using a validated HPLC method.

### LDH release of the everted gut sac

To investigate the viability of the gut sac during the experiment period, the release of LDH, a cytosolic enzyme, was monitored^34^. Its concentration in the incubation medium of mucosal side was measured from 0 min to 240 min according to the protocols of the LDH kit.

### Glucose transport across the everted gut sac

Glucose is actively transported in the small intestine. Healthy and metabolically active sacs will concentrate glucose in the serosal medium and will maintain this gradient. So glucose concentrations were measured in both mucosal and serosal sides for 4 h period of the experiment, their ratios were calculated and compared to the control group to verify the viability and integrity of the gut sac^35^. The concentrations of glucose were measured by a glucose commercial test kit from Rongsheng Biotech Co. Ltd. (China).

### *In situ* single-pass intestine perfusion in rats

Single-pass intestine perfusion studies were performed in rats with some modifications^23^,^34^,^36^. In brief, rats were fasted for 16–18 h (free access to water) prior to the experiment, and anesthetized by intraperitoneal injection of urethane (30 mg/kg), and supplemental doses of anesthetic were given as necessary^37^. After being placed on a heated pad to maintain normal body temperature, a midline incision was made on the abdomen. The biliary duct was ligated, then whole small intestine was isolated and gently flushed with saline (37 °C). Silicone tubes were inserted into the proximal duodenum and terminal ileum and attached to a peristaltic pump (Shanghai Huxi Analysis Instrument Factory, China). Arranged in a uniform S or multi-S pattern to avoid kinks, the small intestine was returned to the abdominal cavity to maintain its viability without disrupting blood vessels. The exposed area was covered with gauze, and saline (37 °C) was applied to keep it warm and moist during the experiment. The intestine was flushed with blank Kreb-Ringer’s buffer (37 °C) until the effluent perfusates were clear.

### Effects of DTX-TPGS-PN on DTX transepithelial permeation

To investigate the effects of DTX-TPGS-PN niosomes on DTX transepithelial permeation, Krebs-Ringer’s buffer solution containing DTX, DTX-PN niosomes and DTX-TPGS-PN niosomes (DTX concentration of 2 μg/mL) were perfused at a constant flow rate of 0.3 mL/min. The intestinal segment was perfused with test solution for about 30 min to achieve absorption equilibrium and stable outflow rates. Subsequently, the effluent perfusates were collected in 10 min interval for 90 min in pre-weighed 5 mL glass vials with a lid. Samples were frozen immediately and stored at −20 °C until analysis. At the end of the experiments, the segment between two cannulas was excised without dragging and its length was measured using silk thread. The DTX concentrations were measured and the permeability coefficients (P_eff_) values were calculated and compared thereafter.

### Water flux correction and permeability calculation

The effective permeability P_eff_ was calculated to compare the absorption of DTX-TPGS-PN niosomes. The net water flux (NWF) was determined by a gravimetric method^24^. C_cor_, the drug concentration of effluent perfusates which was corrected for water flux, was calculated according to the following equation:


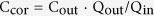


where C_out_ is the concentration of tested drug in the effluent perfusates (μg/mL), Q_in_ and Q_out_ are the inlet and outlet flow rate, respectively, which are adjusted for liquid density (mL/min).

The effective permeability coefficients (P_eff_) were calculated from


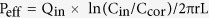


where C_in_ is the concentration of tested drug in the influent perfusate, 2πrL is the area of the mass transfer surface (cm^2^) within the intestinal segment which is assumed to be a cylinder area.

### Pharmacokinetic studies of DTX-TPGS-NP

Pharmacokinetic study was performed in healthy male SD rats. The rats were randomly assigned into four groups (5 rats per group). The pharmacokinetic properties of DTX-TPGS-NP were compared with DTX-NP and a marketed DTX injection (Jiang Su Heng Rui Medicine Co. LTD). The DTX injection was administered intravenously at a dose of 20 mg/kg. Besides, the DTX injection, DTX-PN and DTX-TPGS-PN niosomes were administered orally at a dose of 20 mg/kg. Whole blood samples were collected by retro-orbital puncture into heparinized tubes at 0.25, 0.5, 1, 2, 4, 8, and 12 hours following oral administration and at 0.033, 0.083, 0.25, 0.5, 1, 2 and 4 hours following intravenous administration. The plasma samples obtained were immediately centrifuged at 10,000 rpm for 3 minutes, and 100 μl of plasma was mixed with 300 μl paclitaxel (50 ng/mL) as the internal standard. The supernatant was collected after centrifugation at 12,000 rpm for 10 minutes, and mixed with 100 μL deionized water for the drug content analysis by HPLC-MS (API-3000, Applied Biosystems, USA).

The pharmacokinetic parameters were calculated using a non-compartmental model by the Drug and Statistics (DAS) software (version 2.1.1, Mathematical Pharmacology Professional Committee, China). The absolute bioavailability of DTX after oral administration compared to the intravenous injection was defined as follow:


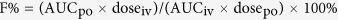


AUC represents the area under the curve, dose_iv_ stands for the dose of intravenous injection, dose_po_ stands for the dose of oral administration.

### Cytotoxicity assay

To evaluate the antitumor efficacy of the prepared DTX-loaded nanoparticles, *in vitro* cellular cytotoxicity on MCF-7 and MDA-MB-231 cells was evaluated by CCK-8 assay. MCF-7 Cells were seeded at a density of 5 × 10^3^ cells/well in 96-well flat bottomed plates, and allowed to adhere overnight. The cells were washed twice with PBS and incubated for 24 h with various concentrations of DTX solution, blank PN, blank TPGS-PN, DTX-NP and DTX-TPGS-NP niosomes. Then cells were washed twice with PBS to eliminate the remaining drugs. Twenty microliters of CCK-8 solution was added to each well and the cells were incubated for 2 h at 37 °C. The absorbance at 450 nm was measured with a microplate reader (Spectra Max190, Molecular Devices, USA).

### Antitumor effect of DTX-TPGS-NP on MCF-7 tumor-bearing mice

The antitumor effect of DTX-TPGS-NPs was investigated on a MCF-7 breast cancer xenograft model. The female nude mice of 4–6 weeks old were subcutaneously injected with 0.1 mL of cell suspension containing 2 × 10^6^ MCF-7 cells in the right flank without estrogen supplement^38^. When the tumors grew to approximately 50–80 mm^3^, the mice were assigned randomly into seven groups (8 mice per group): NS group (control); TPGS-PN group (p.o., 20 mg/kg); DTX group (p.o., 20 mg/kg); marketed DTX injection group (i.v., 10 mg/kg); DTX-PN group (p.o., 20 mg/kg);. DTX-TPGS-PN group (p.o., 10 mg/kg, 20 mg/kg, respectively). The mice were administrated intravenously or orally once every 3 days for 15 days. For tumor growth inhibition study, tumor size and body weight were measured every 3 days during the experimental period. The tumor volume was calculated based on the equation (a × b^2^)/2, where a and b were the length of the major axis and minor axis, respectively. On day 18, the mice were sacrificed and the tumor in each group was collected for further analysis.

The inhibition ratio was calculated by the following formula:


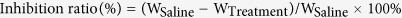


W_Saline_ and W_Treatment_ stand for the tumor weight for saline group and treatment group, respectively.

### Statistical analysis

All the data in this study were analyzed by SPSS 19.0 (SPSS Inc., Chicago). Data are expressed as mean ± SD. For values that were normally distributed, direct comparison between two groups was conducted by Independent Sample’s T test. P value of <0.05 was considered statistically significant.

## Additional Information

**How to cite this article:** Liu, H. *et al*. Improved Bioavailability and Antitumor Effect of Docetaxel by TPGS Modified Proniosomes: *In Vitro* and *In Vivo* Evaluations. *Sci. Rep.* **7**, 43372; doi: 10.1038/srep43372 (2017).

**Publisher's note:** Springer Nature remains neutral with regard to jurisdictional claims in published maps and institutional affiliations.

## Figures and Tables

**Table 1:** Pharmacokinetic parameters of DTX in plasma followed by intravenous administration of DTX solution and oral administration of DTX-TPGS-PN, DTX-PN niosomes and DTX solution (Mean ± SD, *n* = 3, **P < 0.05, compared to DTX solution).

PK parameters	p.o.			i.v.
	DTX-TPGS-PN	DTX-PN	DTX solution	DTX solution
k(h^−1^)	0.22 ± 0.02	0.33 ± 0.17	0.31 ± 0.10	1.02 ± 0.0007
T_1/2_(h)	3.19 ± 0.23**	2.47 ± 0.99	2.39 ± 0.88	0.68 ± 0.0004
C_max_(ng/mL)	155.67 ± 4.73**	27.43 ± 1.53**	19.83 ± 6.30	—
T_max_(h)	0.5	1	1	—
AUC (ng/mL·h)	796.44 ± 83.93**	184.70 ± 13.05**	109.04 ± 17.74	4699.8 ± 429.22
F(%)	16.94**	3.93**	2.32	—
